# Current gaps in data and health information system in the European Region

**DOI:** 10.1093/eurpub/ckac129.132

**Published:** 2022-10-25

**Authors:** S Davia

**Affiliations:** Data and Digital Health Unit, WHO Regional Office for Europe, Copenhagen, Denmark

## Abstract

When the SDGs were adopted in 2015, it was already clear that major issues regarding data availability would need to be tackled to enable comprehensive and policy-relevant monitoring and reporting. The SDG Report 2019 found that most MS were still not regularly collecting data for more than half of the global indicators. The measurement framework (MF) of the EPW builds on the SDGs. Also here, important data gaps for the included health-related SDG indicators exist. These gaps relate, e.g., to coverage of treatment for substance use disorders and the proportion of women with satisfied family planning needs. In addition, the MF exposed data gaps for several other areas that are highly relevant for the WHO European Region, such as health inequalities, intersectoral action for health, ageing populations and mental health. To help overcome these gaps, the EPW MF includes a development list. This list contains 20 indicator areas for which either no well-defined measures have yet been included in Region-wide international data collections or where data are available only for a limited number of MS. It will serve as a priority list for developmental work on indicators in the European Region in the coming years. Data gaps as described above are not stand-alone problems but are linked to wider issues with the functioning of HISs. In the European Region, the main HIS challenges relate to limited resources and capacity; insufficient coordination and collaboration, leading to fragmentation and problems with interoperability; lack of central governance; and limited use of health information for decision-making. In this presentation, we will give an overview of the main gaps in data and HISs in the European Region and explain the roadmap for the implementation of the EPW MF, which will be an important tool to achieve sustainable improvement of data availability and quality for key indicators. Tools to improve HIS performance overall will be addressed in the next presentation.

